# Varicose veins treatment in England: population-based study of time trends and disparities related to demographic, ethnic, socioeconomic, and geographical factors

**DOI:** 10.1093/bjsopen/zrac077

**Published:** 2022-07-07

**Authors:** Jonathan A Michaels, Shah Nawaz, Thaison Tong, Paul Brindley, Stephen J Walters, Ravi Maheswaran

**Affiliations:** School of Health and Related Research, University of Sheffield, Sheffield, UK; Sheffield Vascular Institute, Sheffield Teaching Hospitals NHS Foundation Trust, Sheffield, UK; School of Health and Related Research, University of Sheffield, Sheffield, UK; Department of Landscape Architecture, University of Sheffield, Sheffield, UK; School of Health and Related Research, University of Sheffield, Sheffield, UK; School of Health and Related Research, University of Sheffield, Sheffield, UK

## Abstract

**Background:**

Varicose vein (VV) treatments have changed significantly in recent years leading to potential disparities in service provision. The aim of this study was to examine the trends in VV treatment in England and to identify disparities in the provision of day-case and inpatient treatments related to deprivation, ethnicity, and other demographic, and geographical factors.

**Method:**

A population-based study using linked hospital episode statistics for England categorized VV procedures and compared population rates and procedure characteristics by ethnicity, deprivation quintile, and geographical area.

**Results:**

A total of 311 936 people had 389 592 VV procedures between 2006/07 and 2017/18, with a further 63 276 procedures between 2018/19 and 2020/21. Procedure rates have reduced in all but the oldest age groups, whereas endovenous procedures have risen to more than 60 per cent of the total in recent years. In younger age groups there was a 20–30 per cent reduction in procedure rates for the least-deprived compared with the most-deprived quintiles. Non-white ethnicity was associated with lower procedure rates. Large regional and local differences were identified in standardized rates of VV procedures. In the most recent 5-year interval, the North-East region had a three-fold higher rate than the South-East region with evidence of greater variation between commissioners in overall rates, the proportion of endovenous procedures, and policies regarding bilateral treatments.

**Conclusions:**

There are substantial geographical variations in the provision of treatment for VVs, which are not explained by demographic differences. These have persisted, despite the publication of guidelines from the National Institute for Health and Care Excellence, and many commissioners, and providers would seem to implement policies that are contrary to this guidance. Lower rates of procedures in less-deprived areas may reflect treatments carried out in private practice, which are not included in these data.

## Teaser

There have been significant changes in the available treatments for people with varicose veins. Endovenous and surgical treatments for people with symptomatic varicose veins are clinically effective and cost-effective, and currently recommended in National Institute for Health and Care Excellence (NICE) guidelines; however, there remain significant geographical disparities in access to varicose vein treatments that are not explained by socioeconomic, ethnic, or demographic factors. NICE guidelines seem to have done little to address such disparities, with many commissioners implementing policies that seem to be contrary to this guidance.

## Introduction

Chronic venous insufficiency and varicose veins (VVs) are common conditions, although estimates of the prevalence in Western Europe and the USA vary widely^1^. In the UK, the Edinburgh Vein Study suggested an age-adjusted prevalence of chronic venous insufficiency of 9 per cent in men and 7 per cent in women, and a prevalence of trunk varices of 40 per cent in men and 32 per cent in women^2^. Over the past 15 years there have been many changes affecting the surgical management of VVs in England. Policy and organizational changes have included the centralization of vascular services^3^, changes in the commissioning arrangements^4^, and the introduction of independent sector treatment centres^5^. Changes in clinical practice have resulted from the introduction of new endovascular technologies^6^, emerging research evidence from large, randomized studies^7–10^, and the publication of National Institute for Health and Care Excellence (NICE) guidance^11^.

A previous study of the provision of vascular services in England has identified some of the changes that have resulted from the centralization of services for major vascular surgery^3^; however, it also suggested that this has had knock-on effects, in that it has influenced the delivery of investigations and more minor procedures that have tended to follow the centralization of major surgery, potentially affecting the local availability of these services. In the context of rapid changes in practice and policy, and varying local guidance, there is a risk that barriers to referral and treatment will lead to disparities in access, clinical practice, and outcomes, based upon demographic, socioeconomic, ethnic, or geographical factors.

The principal aim of this population-based study that has been funded by the National Institute for Health Research (NIHR) as a Programme Development grant (https://fundingawards.nihr.ac.uk/award/NIHR202042), is to carry out further detailed analysis of routinely collected data to examine the trends in vascular treatment in England and to identify disparities in service provision, practice, and outcomes related to deprivation, ethnicity, and other demographic, and geographical factors. This paper presents the findings in respect to the provision of day-case and inpatient treatments for VVs.

## Methods

A data extract of hospital episode statistics (HES) from NHS Digital, covering the financial years from 2006/7 to 2017/8, was processed, and classified as part of a larger study of vascular services. The methods for categorization and cleaning of data have been described elsewhere^3^. Day-case and inpatient admissions for VV treatments were identified within the data extract and categorized as open surgery, endovenous laser treatment (EVLT), radiofrequency ablation (RFA), sclerotherapy, and avulsions only. A pseudo-anonymized patient identifier was used to link data to identify readmissions and repeat procedures.

Patients were classified based upon sex, broad age groups (10-year bands such as 15 to 24, 25 to 34, to more than 85), broad ethnicity categories (as used in reporting the 2011 census results), and geographical, and socioeconomic categories, based upon lower layer super output areas (LSOA). The income domain from the Index of Multiple Deprivation (IMD) 2010 was used as the indicator of socioeconomic deprivation at the LSOA level (Communities and Local Government 2011). The IMD is the national index of deprivation widely used by government agencies in England.

Population data for most analyses were based upon Office for National Statistics data mid-year estimates by 10-year age group, sex, and deprivation quintile, using LSOA population estimates. For calculation of population rates, cases, and population estimates were restricted to LSOAs that remained consistent across the time interval.

Population data relating to ethnicity were only available by broader age group and LSOA level for the 2011 census year. Due to concerns about changing population demographics affecting projections for other years, estimates of population rates by ethnicity were limited to the 5-year interval 2009 to 2013. Expected procedure rates and indirectly standardized relative rates were calculated for ethnic groups and geographical areas, standardized for age, sex, and deprivation quintile use the R PHEindicatormethods package^12^.

Geographical factors were considered based upon English regions and Clinical Commissioning Group (CCG) catchment areas, using the 2021 definitions and population estimates from the LSOA data, mapped through the NHS postcode directory^13^.

Overall time trends were supplemented by publicly available data using four-digit OPCS code for primary procedure for years beyond the available episode-level dataset^14^. The same classification system was used for categorizing cases based upon OPCS code, except that only primary diagnosis was available, so cases in which a higher priority procedure occurred as a secondary diagnosis may have been classified differently. Data on VV procedures in private practice were identified for the last complete pre-pandemic year (2019–2020) from the Private Healthcare Information Network (PHIN) and regional estimates obtained by mapping provider postcodes to NHS regions using the NHS postcode directory^13^.

## Results

The analysis of the HES extract identified 311 936 people undergoing a total of 389 592 VV procedures between 2006/07 and 2017/18. A further 63 276 procedures between 2018/19 and 2020/21 were identified from publicly available data based upon four-digit OPCS codes.

### Overall trends

**Fig. 1:**
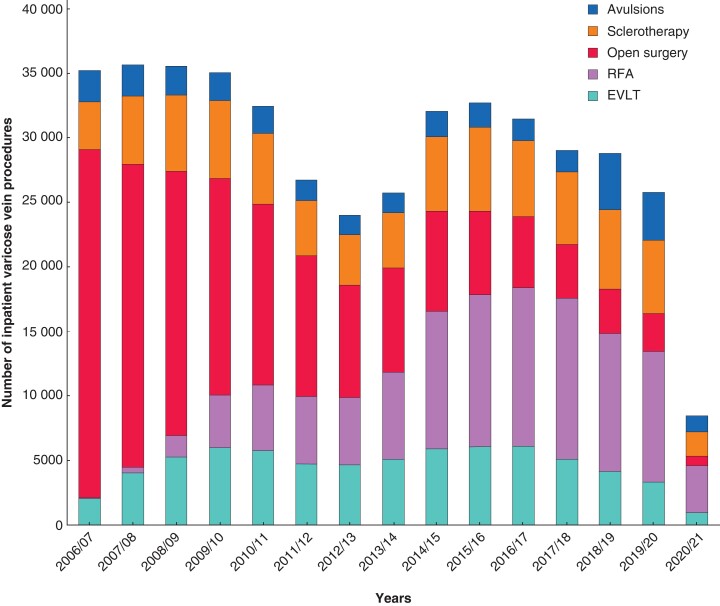
Trends in varicose vein procedures in England from 2006 to 2021—number of inpatient varicose vein procedures (data from 2018 using public data based upon primary procedure) RFA, radiofrequency ablation; EVLT, endovenous laser treatment.

**Fig. 2:**
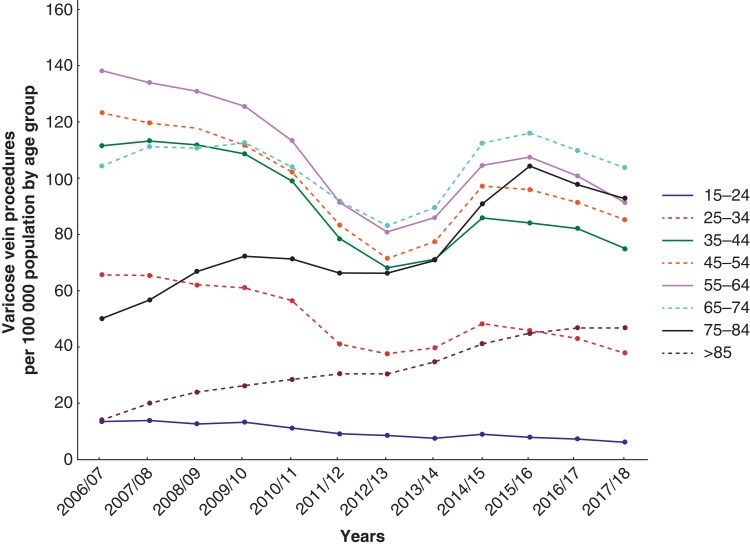
Trends in varicose vein procedures in England from 2006 to 2018—rate per 100 000 population by age (years) group

From 2006/07 to 2009/10 there were more than 35 000 elective day-case or inpatient admissions per year for VV treatments, with a marked dip and recovery around 2012/13, followed by a more gradual decline and then a marked reduction related to the pandemic in 2020/21. Over this time, treatment methods have changed, with fewer open procedures and more endovenous methods, particularly RFA (*Fig. 1*). The highest population rates were in the early years in the 55–64 age group at 138 per 100 000 patient years, with a marked trend to increasing rates in those older than 75 years (from 40 to 87 per 100 000 per year) but reducing rates in all other age groups (*Fig. 2*).

*Table 1* provides details of the changes in demographics and procedures carried out. Trends have included reduced waiting times, an increase in day-case procedures, a reduction in the proportion of bilateral procedures, increased repeat procedures within a year, greater proportions of males, and older patients. The number of procedures carried out where there was a diagnosis of venous ulcer increased three-fold, to represent nearly 7 per cent of cases. In the most recent year of detailed data (2017–2018) endovenous treatment (EVLT or RFA) accounted for 60.4 per cent of cases, open surgery for 14.5 per cent, sclerotherapy for 19.2 per cent, and avulsions alone for 5.8 per cent.

**Table 1:** Trends in the type of procedure and demographics of patients undergoing inpatient varicose vein treatments

	2006/07	2007/08	2008/09	2009/10	2010/11	2011/12	2012/13	2013/14	2014/15	2015/16	2016/17	2017/18
**Total procedures**	35 241	35 681	35 566	35 076	32 461	26 738	24 017	25 749	32 060	32 720	31 481	29 038
Age (years)—median (i.q.r.)*	50 (39–60)	50 (39–61)	50 (40–61)	50 (40–62)	51 (40–63)	52 (41–64)	52 (41–65)	53 (42–65)	53 (42–66)	54 (43–67)	54 (43–67)	54 (43–68)
**Female (%)**	64.5	64.3	64.0	63.0	62.6	60.2	58.4	57.5	59.4	58.2	56.6	55.9
Waiting time (weeks)—median (i.q.r.)*	125 (65–171)	88 (50–132)	63 (37–96)	65 (38–98)	67 (37–105)	65 (36–105)	64 (35–103)	67 (37–107)	71 (40–113)	68 (38–108)	73 (41–119)	76 (41–126)
**Endovenous procedures (%)**	6.0	12.5	19.4	28.7	33.5	37.3	41.1	45.9	51.6	54.6	58.4	60.5
**Day-case procedure (%)**	74.3	79.2	83.3	86.6	88.1	89.4	91.3	92.1	94.1	95.0	95.3	96.2
**Reoperation within <1 year (%)**	9.5	11.2	12.5	13.3	12.8	13.7	14.1	15.1	15.0	15.1	15.1	NA
**Bilateral procedures (%)**	17.3	16.6	14.5	14.2	13.8	14.0	13.9	13.2	13.4	12.1	12.1	11.9
**Venous ulcer diagnosis (%)**	1.7	2.2	2.5	2.8	3.6	5.2	5.5	5.7	5.1	5.7	5.6	6.9
**Rate (per 100 000 person years)**	71.35	71.74	70.99	69.57	63.91	52.23	46.62	49.67	61.37	62.16	59.34	54.73
**Proportion non**-**white (%)**	6.2	6.9	7.2	7.4	8.1	8.5	8.5	8.6	9.1	9.2	10.0	11.4

* i.q.r., interquartile range.

### Ethnic and socioeconomic disparities

Deprivation data based upon LSOA data were available for 96.4 per cent of English residents and ethnicity was available from HES data for 94.4 per cent of VV procedures. Greater deprivation was associated with a trend towards younger age at the time of the procedure, but other demographic and procedural characteristics were similar across deprivation quintiles (*Table 2*). Overall rates of procedures were similar across quintiles for those aged more than 65 years, but in younger age groups there was a marked trend towards lower rates for the less-deprived quintiles, with 20 to 30 per cent fewer procedures in the least, compared with the most-deprived quintiles.

**Table 2:** Procedure details and demographics of patients undergoing inpatient varicose vein treatments by deprivation quintile (2013/14 to 2017/18)

Deprivation quintile (1 most deprived)	1	2	3	4	5
**Total procedures**	30 370	31 055	31 384	30 403	27 836
Age (years)—median (i.q.r.)*	49 (38–61)	52 (41–65)	55 (43–68)	56 (44–68)	57 (45–69)
**Female (%)**	58.5	57.4	57.6	56.8	57.4
**Endovenous procedures (%)**	56.5	55.5	53.5	53.1	53.2
**Day-case procedure (%)**	95.1	94.7	94.7	94.4	94.0
**Reoperation within <1 year (%)**	13.8	13.8	13.9	13.8	13.2
**Bilateral procedures (%)**	11.9	11.9	12.6	13.0	13.2
**Venous ulcer diagnosis (%)**	5.1	6.1	6.5	5.7	5.5
**Rate (per 100 000 person years) more than 65 years**	80.98	85.14	87.65	85.73	85.46
**Rate (per 100 000 person years) 35–64 years**	109.15	104.95	100.62	95.06	85.35
**Rate (per 100 000 person years) under 35 years**	35.14	32.65	30.11	28.36	24.13
**Non-white ethnicity (%)**	18.9	12.7	7.2	4.7	4.4

* i.q.r., interquartile range.

Ethnicity and deprivation were closely related. Black ethnicity was associated with greatest deprivation, with nearly 80 per cent of those undergoing treatment living in areas in the lowest two deprivation quintiles. The proportion recorded as non-white ethnicity rose from 6.2 per cent in 2006/07 to 11.4 per cent in 2017/18. On average, those with non-white ethnicity were younger, had lower rates of VV procedures, apart from those recorded as ‘other’ ethnicity, and were more likely to undergo endovenous procedures, whereas those with black ethnicity were more likely to have an ulcer diagnosis (*Table 3*).

**Table 3:** Procedure details and demographics of patients undergoing inpatient varicose vein treatments by ethnicity (2006/07 to 2017/18)

Ethnicity	White	Asian	Black	Mixed	Other	Unknown
**Total procedures**	126 136	6300	1742	781	2369	6713
Age (years)—median (i.q.r.)*	52 (41–64)	46 (37–57)	47 (40–56)	43 (34–52)	45 (36–55)	48 (39–58)
**Female (%)**	61.6	53.1	62.0	58.9	57.3	50.6
**Endovenous procedures (%)**	35.4	43.8	47.3	41.4	47.4	42.7
**Day-case procedure (%)**	88.9	90.5	90.5	90.4	91.7	92.1
**Reoperation within <1 year (%)**	13.9	15.3	12.3	11.5	13.8	10.4
**Bilateral procedures (%)**	13.9	13.6	11.0	14.3	13.0	15.1
**Venous ulcer diagnosis (%)**	4.4	4.5	6.7	2.8	3.4	3.4
Income deprivation score—median (i.q.r.)*	0.11 (0.06–0.19)	0.19 (0.12–0.3)	0.24 (0.15–0.32)	0.17 (0.1–0.27)	0.18 (0.1–0.28)	0.11 (0.06–0.19)
**Rate (per 100 000 person years)^†^**	69.93	42.97	27.70	25.37	122.81^‡^	NA

* i.q.r., interquartile range. ^†^Population rates based upon years 2008/09 to 2012/13. ^‡^May be overestimated due to differences in designations of ethnicity. NA, not available.

### Geographical disparities

**Fig. 3:**
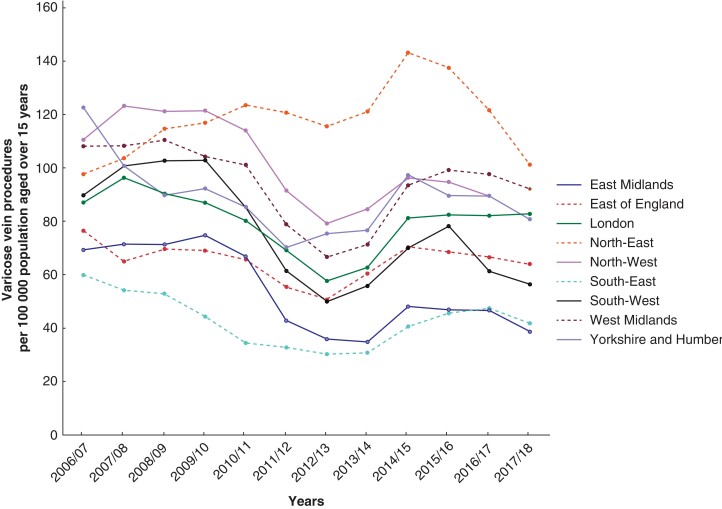
Crude procedure rate by region (procedures per 100 000 population aged more than 15 years)

There are considerable regional disparities in both the rate and characteristics of VV procedures. Crude annual rates for procedures in adults vary from 29.4 per 100 000 per year (South-East region in 2013/14) to 136.5 per 100 000 per year (North-East region in 2014/15) with annual rates varying by up to 400 per cent between regions (*Fig. 3*). These differences persist after standardization for age, sex, and deprivation (*Table 4*). Although all regions have substantially reduced the rate of open procedures over the years, this has occurred at different rates, with London, the South-West and West Midlands carrying out fewer than 50 per cent of cases by open surgery by 2008/09, whereas in the East of England and Yorkshire and Humber this extent of reduction occurred four years later.

**Table 4:** Rates of varicose veins procedures per 100 000 population by region, standardized for age, sex, and deprivation

	2006/07	2007/08	2008/09	2009/10	2010/11	2011/12	2012/13	2013/14	2014/15	2015/16	2016/17	2017/18
**East Midlands**	66.4	68.1	67.4	71.5	64.4	40.4	34.3	32.3	45.6	43.6	43.8	36.6
**East of England**	74.2	62.9	67.2	66.1	63.3	53.3	48.8	58.1	66.9	64.7	63.8	60.0
**London**	87.5	96.7	91.2	87.7	80.3	68.5	57.5	62.0	79.4	80.4	79.9	80.5
**North-East**	90.1	95.2	105.2	108.8	114.5	112.5	107.9	114.0	132.0	127.5	108.6	92.0
**North-West**	106.6	120.0	117.8	117.5	110.2	89.0	77.0	82.0	93.3	91.2	85.6	77.5
**South-East**	59.5	53.6	51.9	43.6	34.1	32.5	30.0	29.8	39.3	44.6	46.4	40.7
**South-West**	85.8	97.0	98.7	98.1	81.5	59.0	47.9	53.2	65.9	74.6	57.7	53.0
**West Midlands**	103.5	104.2	106.3	100.6	98.0	76.1	64.1	68.6	89.4	94.9	93.1	88.0
**Yorkshire and Humber**	118.6	96.8	87.1	89.5	82.6	68.2	73.0	74.4	93.5	85.9	84.9	77.3

i.q.r., interquartile range.

The use of different technologies varied between regions. Other differences in practice, include different rates of sclerotherapy, ranging from less than 10 per cent to more than 40 per cent and differences in the proportion of bilateral and repeat procedures (*Table 5*).

**Table 5:** Procedure details and demographics of patients undergoing inpatient varicose vein treatments by region (2013/14 to 2017/18)

Region	East Midlands	East of England	London	North-East	North-West	South-East	South-West	West Midlands	Yorkshire and Humber
**Mean procedures per year**	1514	3028	5072	2473	5017	2814	2720	3990	3581
Age (years)—median (i.q.r.)*	56 (45–68)	55 (43–68)	51 (40–64)	52 (42–64)	53 (42–66)	57 (44–69)	60 (47–70)	54 (43–67)	50 (40–63)
**Female (%)**	50.5	57.6	58.8	61.6	59.9	54.2	53.4	56.0	60.2
**Endovenous procedures (%)**	48.7	51.0	66.1	54.6	56.1	56.1	37.5	53.3	53.4
**Day-case procedure (%)**	90.7	92.7	95.0	95.7	94.8	93.6	95.4	96.2	94.6
**Reoperation within <1 year (%)**	8.0	14.1	13.7	22.8	13.8	11.8	17.9	12.8	8.5
**Bilateral procedures (%)**	16.1	11.6	11.5	5.5	11.9	13.7	11.2	12.2	19.3
**Venous ulcer diagnosis (%)**	9.0	5.2	5.2	3.4	3.1	10.2	11.9	6.0	2.8
**Rate (per 100 000 person years)**	33.8	52.2	61.2	99.2	72.2	32.8	52.0	72.1	69.0
**Private procedures 2019-20 (from PHIN)**	823	1937	3019	351	905	4191	1729	954	709

* i.q.r., interquartile range; PHIN, Private Healthcare Information Network.

Consideration of geographical differences based upon CCG catchment populations since 2013/14 shows even more marked disparities in population rates and the proportion undergoing endovenous procedures (*Table S1*). Other differences include the rate of bilateral procedures, which varies from less than 5 per cent to more than 30 per cent, and the rate of redo procedures, which seems inversely related to this. In recent years, the centralization of vascular services has resulted in only about half of CCGs having a local specialist vascular service. Comparing those with and without such a local service suggested a higher rate of VV procedures in those CCGs without such a service (*Table 6*).

**Table 6:** Procedure rates per 100 000 population (over 15 years) for CCG areas with and without a vascular service within the CCG area

	Non-vascular	Vascular
**2006/07**	99.4	87.4
**2007/08**	97.2	88.4
**2008/09**	101.3	86.6
**2009/10**	103.6	83.5
**2010/11**	106.1	75.0
**2011/12**	90.1	61.4
**2012/13**	78.3	54.5
**2013/14**	80.6	57.9
**2014/15**	93.5	71.5
**2015/16**	90.0	74.5
**2016/17**	85.1	70.8
**2017/18**	77.6	65.9

CCG, Clinical Commissioning Group.

## Discussion

The intention of this study was to identify and characterize trends and disparities in the provision of treatment for VVs in England. The results demonstrate significant changes over the past 15 years, with a reduction in overall rates of procedures, increased use of endovenous methods, and increasing treatment of older people, and those with venous ulceration.

Some disparities related to socioeconomic and ethnic factors have been identified, including lower rates of procedures in those of non-white ethnicity and a trend towards lower rates of treatment among younger people in areas of lower income deprivation. However, the greatest disparities in treatment seem to relate to regional and local practices in respect to the selection of patients for treatment, the treatment modalities offered, and local practice regarding bilateral or staged procedures for those with VVs affecting both legs. The staging of procedures may relate to differences in practice due to the greater use of procedures under local anaesthesia, although there may also be perverse financial incentives that encourage the staging of bilateral procedures.

The NICE guideline^11^, published in 2013, recommended that those with symptomatic VVs should be referred to a vascular service and that those with confirmed VVs and truncal reflux should be offered endovenous treatment (EVLT or RFA) as a first choice. Although previous publications have suggested some changes in practice in response to the guideline^15,16^, a previous review has suggested that there are very different policies on the commissioning of VV treatments, which are often far more restrictive that suggested in the NICE guidance^17^. The data suggest that these differences in commissioning policy are resulting in considerable regional and local differences in the implementation of this guidance and availability of appropriate VV treatments.

This is a large and comprehensive study of a national data set for all VV procedures over an interval of considerable change in practice. As with any study based upon routine data there are several limitations. Coding of procedures and other information is restricted and may change over time. In identifying and classifying procedures there may be conflicting or non-specific codes, and the algorithms used to deal with such circumstances have been developed and described elsewhere^3^. Some codes may cover several different procedures, such as those for sclerotherapy, which may be for axial veins or tributary treatment. There are difficulties in determining population rates based upon ethnicity and deprivation, with limited data available for detailed population estimates that allow categorization by age, sex, ethnicity, and deprivation. For this reason, estimates of rates by ethnicity were limited to the interval around the 2011 census. There may also be differences in categorization of ethnicity based upon census data *versus* those available in HES data, due to coding discrepancies and differences in collection methods. This may explain the apparently high estimated procedure rates for ‘other’ ethnicities.

There are also difficulties in considering geographical factors, due to changing, and inconsistent definitions of geography. Approximately 3.6 per cent of LSOA areas were excluded due to such changes in definitions. Although the HES data included health authority areas, these are no longer used in practice and a decision was made to define geography based upon 2021 CCG areas. This required remapping of population and HES data based upon postcodes and LSOA data.

Another potential limitation in the interpretation of the data relates to the lack of information regarding private practice. Detailed information about private practice, for comparison with NHS data, is not available. It has previously been estimated that approximately 20 per cent of VV procedures are carried out in the private sector^18^; however, recent PHIN data suggest that this is now nearer to 35 per cent and varies by region, accounting for nearly 60 per cent of cases in the South-East region, which seems to have the most restrictive guidance, compared with only 12 per cent in the North-East. If a significant proportion of cases are treated in the private sector, then this may be partly responsible for the lower NHS procedure rates that were observed in less-deprived areas.

Other limitations relate to the lack of available information regarding potentially important risk factors that may be related to geography, deprivation, and ethnicity, such as occupation, obesity, and smoking; however, the extent of local and regional variation seems unlikely to be explained by such factors and it seems more likely to relate to local referral and treatment policies. This is borne out by published local guidelines, with many CCGs limiting referral guidance to those with skin changes^19,20^, some only considering those with previous approval for ulceration or bleeding^21^, while others seem to fully implement the NICE guideline^22^. These geographical differences in policy have been well documented in a previous review^17^. Part of the rationale for the formation of NICE was the need to address geographical variations in practice, colloquially described as ‘postcode prescribing’^23^; however, the NICE guideline seems to have done little to address long-standing regional variations^24^ in access to VV treatments.

VV treatments have been a long-standing problem for commissioners of services. Historically, they were often considered of low priority, resulting in long waiting lists. With targets to reduce waiting lists, many commissioners found ways to manage demands, resulting in significant reduction in procedure numbers. Despite the evidence that, in NHS terms, treatment is highly cost-effective, there is no ear-marked payment or funding directive. Thus, commissioners faced with financial pressures, may allocate resources to less cost-effective services based on urgency, perceived clinical priority, or mandatory commitments. Without financial incentives or monitoring and policing of guideline recommendations, it is unlikely that such guidance will be consistently implemented, a situation that may be exacerbated by perverse incentives relating to a significant rise in private practice.

Concerns that centralization of vascular services might reduce access to VV treatment for those without a local vascular service seem unfounded. The evidence suggests the converse, that areas without a local vascular service have higher rates of VV procedures, perhaps reflecting a potential issue with a lack of capacity in centralized services, leading to more-stringent restrictions on minor procedures that compete for resources with more-urgent vascular conditions.

Studies have found the treatment of symptomatic VVs to be both clinically effective and highly cost-effective in the terms usually used by the NHS^3,9,25,26^. Despite national guidance from NICE having accepted this, and having recommended interventional treatment for symptomatic people, the condition is considered relatively minor and therefore has become an easy target for rationing. This has resulted in variable access to services, depending upon local commissioning and provider policies, potentially increasing both geographical and socioeconomic inequalities by restricting treatment to those who can access private services. This situation is likely to be further exacerbated by the limitation of routine surgery and backlogs caused by the COVID-19 pandemic.

## Supplementary Material

zrac077_Supplementary_Data

## Data Availability

All code used in the analysis will be available from the authors on reasonable request. The underlying data from HESs are available through NHS Digital subject to an appropriate data-sharing agreement.
