# Supplementary material for: Varicose veins treatment in England: population-based study of time trends and disparities related to demographic, ethnic, socioeconomic, and geographical factors
Source: BJS Open. 2022 Jul 7;6(4):zrac077. doi: 10.1093/bjsopen/zrac077 (PMC9260184; doi:10.1093/bjsopen/zrac077)
Supplement: zrac077_Supplementary_Data [file zrac077_supplementary_data.docx]

Table S1. Procedure details and demographics of patients undergoing inpatient varicose vein treatments by Clinical Commissioning Group area of residence (2013/14 to 2017/18).

| **CCG** | **ccg_name** | **patients** | **observed procedures** | **Crude procedure rate per 100,000** | **expected procedures** | **Standardised rate ratio** | **Age - median (IQR)** | **Female (%)** | **Endovenous procedures (%)** | **Day case procedure (%)** | **Reoperation < 1 year (%)** | **Bilateral procedures (%)** | **Venous ulcer diagnosis (%)** |
| --- | --- | --- | --- | --- | --- | --- | --- | --- | --- | --- | --- | --- | --- |
| 10R | NHS Portsmouth CCG | 100 | 111 | 13.2 | 527 | 0.21 (0.17-0.25) | 52 (40-67) | 44.1% | 17.1% | 91.0% | 7.2% | 8.1% | 40.5% |
| D9Y0V | NHS Hampshire, Southampton and Isle of Wight CCG | 777 | 886 | 14.3 | 4312 | 0.21 (0.19-0.22) | 60 (45-70.8) | 51.2% | 21.6% | 79.7% | 9.9% | 10.8% | 17.8% |
| 04C | NHS Leicester City CCG | 235 | 255 | 20.0 | 844 | 0.30 (0.27-0.34) | 52 (40-60) | 40.0% | 29.0% | 81.6% | 3.9% | 16.5% | 4.7% |
| 15C | NHS Bristol, North Somerset and South Gloucestershire CCG | 661 | 754 | 21.5 | 2343 | 0.32 (0.30-0.35) | 54 (42-69) | 52.4% | 34.5% | 91.8% | 10.6% | 17.0% | 9.7% |
| 15A | NHS Berkshire West CCG | 406 | 441 | 23.8 | 1223 | 0.36 (0.33-0.40) | 55 (43-66) | 49.4% | 37.4% | 92.1% | 7.0% | 23.6% | 7.7% |
| 04V | NHS West Leicestershire CCG | 351 | 388 | 24.4 | 1075 | 0.36 (0.33-0.40) | 55 (44-66) | 42.5% | 43.8% | 87.4% | 5.9% | 19.8% | 9.3% |
| 02X | NHS Doncaster CCG | 318 | 337 | 27.2 | 901 | 0.37 (0.34-0.42) | 48 (39-58) | 64.7% | 2.1% | 95.3% | 5.3% | 2.7% | 0.6% |
| 03W | NHS East Leicestershire and Rutland CCG | 309 | 337 | 25.4 | 920 | 0.37 (0.33-0.41) | 58 (48-68) | 45.1% | 34.4% | 86.6% | 5.0% | 16.6% | 6.5% |
| 02Q | NHS Bassetlaw CCG | 125 | 136 | 28.4 | 352 | 0.39 (0.32-0.46) | 50.5 (38-60) | 64.7% | 8.1% | 95.6% | 7.4% | 5.9% | 2.9% |
| 06H | NHS Cambridgeshire and Peterborough CCG | 790 | 909 | 27.3 | 2266 | 0.40 (0.38-0.43) | 58 (44-69) | 57.0% | 25.1% | 92.6% | 10.7% | 7.2% | 12.5% |
| 03H | NHS North East Lincolnshire CCG | 165 | 185 | 28.8 | 467 | 0.40 (0.34-0.46) | 49 (40-61) | 50.8% | 62.2% | 92.4% | 8.1% | 13.5% | 2.7% |
| 78H | NHS Northamptonshire CCG | 707 | 801 | 29.8 | 1881 | 0.43 (0.40-0.46) | 60 (49-70) | 50.4% | 43.2% | 87.9% | 9.1% | 24.5% | 9.4% |
| 03K | NHS North Lincolnshire CCG | 188 | 215 | 31.2 | 505 | 0.43 (0.37-0.49) | 52 (44-66) | 56.7% | 53.0% | 92.6% | 10.2% | 13.5% | 1.4% |
| 97R | NHS East Sussex CCG | 701 | 840 | 37.2 | 1679 | 0.50 (0.47-0.54) | 55.5 (44-69) | 49.5% | 58.1% | 93.3% | 13.3% | 13.0% | 6.8% |
| 07G | NHS Thurrock CCG | 190 | 216 | 35.0 | 429 | 0.50 (0.44-0.58) | 52 (41-64) | 52.3% | 54.2% | 93.1% | 9.3% | 12.0% | 4.6% |
| 06Q | NHS Mid Essex CCG | 471 | 570 | 38.2 | 1051 | 0.54 (0.50-0.59) | 59 (48-71) | 53.9% | 44.9% | 93.2% | 12.8% | 3.5% | 8.6% |
| D4U1Y | NHS Frimley CCG | 918 | 1037 | 36.9 | 1867 | 0.56 (0.52-0.59) | 54 (44-67) | 54.0% | 67.4% | 91.2% | 7.6% | 19.5% | 4.8% |
| 01W | NHS Stockport CCG | 392 | 477 | 40.3 | 845 | 0.56 (0.52-0.62) | 56 (46-66) | 59.1% | 56.6% | 89.7% | 14.3% | 12.2% | 5.5% |
| 91Q | NHS Kent and Medway CCG | 2348 | 2829 | 40.5 | 4949 | 0.57 (0.55-0.59) | 54 (42-66) | 55.7% | 45.7% | 95.0% | 14.1% | 3.5% | 8.1% |
| 99E | NHS Basildon and Brentwood CCG | 350 | 420 | 40.5 | 728 | 0.58 (0.52-0.64) | 54 (43.8-66) | 51.2% | 53.6% | 95.5% | 11.4% | 11.0% | 8.1% |
| 03N | NHS Sheffield CCG | 709 | 847 | 39.2 | 1441 | 0.59 (0.55-0.63) | 49 (39-60) | 54.8% | 44.4% | 97.3% | 13.7% | 12.8% | 2.7% |
| M1J4Y | NHS Bedfordshire, Luton and Milton Keynes CCG | 1249 | 1438 | 41.7 | 2378 | 0.60 (0.57-0.64) | 55 (43-68) | 49.4% | 44.8% | 93.8% | 10.7% | 16.3% | 7.4% |
| 99A | NHS Liverpool CCG | 642 | 756 | 41.9 | 1246 | 0.61 (0.56-0.65) | 54 (44-65) | 54.5% | 69.3% | 97.4% | 11.2% | 9.5% | 4.5% |
| 10Q | NHS Oxfordshire CCG | 817 | 1067 | 41.1 | 1686 | 0.63 (0.60-0.67) | 61 (47-72) | 52.2% | 41.4% | 95.3% | 20.3% | 13.2% | 22.0% |
| 14L | NHS Manchester CCG | 629 | 750 | 40.5 | 1166 | 0.64 (0.60-0.69) | 49 (37-62) | 61.6% | 57.2% | 89.9% | 12.8% | 10.8% | 4.4% |
| 70F | NHS West Sussex CCG | 1354 | 1562 | 46.0 | 2427 | 0.64 (0.61-0.68) | 59 (46-72) | 51.2% | 60.3% | 95.8% | 10.6% | 16.6% | 12.9% |
| B2M3M | NHS Coventry and Warwickshire CCG | 1328 | 1576 | 45.2 | 2370 | 0.66 (0.63-0.70) | 55.5 (45-68) | 49.6% | 54.7% | 96.1% | 12.8% | 8.1% | 4.4% |
| 07H | NHS West Essex CCG | 485 | 560 | 47.2 | 836 | 0.67 (0.62-0.73) | 53 (42-64) | 61.3% | 55.9% | 85.9% | 9.5% | 10.9% | 4.3% |
| 52R | NHS Nottingham and Nottinghamshire CCG | 1611 | 1811 | 46.0 | 2721 | 0.67 (0.64-0.70) | 58 (45-69) | 48.4% | 39.9% | 96.0% | 8.3% | 14.4% | 14.4% |
| 00T | NHS Bolton CCG | 451 | 556 | 50.2 | 787 | 0.71 (0.65-0.77) | 52 (40-66) | 59.0% | 49.6% | 96.2% | 16.4% | 5.9% | 4.3% |
| 11M | NHS Gloucestershire CCG | 1107 | 1268 | 50.7 | 1768 | 0.72 (0.68-0.76) | 56 (44-67) | 55.1% | 3.2% | 92.8% | 11.6% | 12.9% | 4.2% |
| 71E | NHS Lincolnshire CCG | 1401 | 1573 | 52.6 | 2191 | 0.72 (0.68-0.75) | 56 (44-68) | 52.3% | 66.0% | 93.3% | 7.6% | 14.1% | 6.0% |
| 92G | NHS Bath and North East Somerset, Swindon and Wiltshire CCG | 1491 | 1752 | 50.6 | 2373 | 0.74 (0.70-0.77) | 54 (42-66) | 56.0% | 38.8% | 93.9% | 13.1% | 6.2% | 9.4% |
| 15M | NHS Derby and Derbyshire CCG | 1927 | 2169 | 53.6 | 2898 | 0.75 (0.72-0.78) | 55 (44-66) | 53.0% | 55.2% | 87.0% | 8.9% | 16.3% | 8.2% |
| 05W | NHS Stoke on Trent CCG | 460 | 555 | 53.1 | 744 | 0.75 (0.69-0.81) | 56 (43-66.5) | 48.3% | 55.7% | 89.9% | 14.2% | 13.3% | 9.4% |
| 14Y | NHS Buckinghamshire CCG | 958 | 1067 | 51.5 | 1411 | 0.76 (0.71-0.80) | 60 (46-71) | 55.7% | 55.0% | 92.9% | 7.3% | 14.2% | 12.7% |
| 01G | NHS Salford CCG | 401 | 479 | 54.2 | 621 | 0.77 (0.70-0.84) | 51 (41.5-64) | 56.2% | 58.0% | 95.8% | 13.4% | 6.7% | 2.5% |
| 03L | NHS Rotherham CCG | 511 | 605 | 57.3 | 773 | 0.78 (0.72-0.85) | 47 (38-58) | 69.1% | 43.8% | 97.5% | 12.9% | 17.0% | 0.8% |
| 01T | NHS South Sefton CCG | 334 | 375 | 58.3 | 480 | 0.78 (0.70-0.86) | 59 (47-69) | 52.8% | 71.7% | 96.8% | 8.5% | 11.2% | 2.4% |
| 11J | NHS Dorset CCG | 1482 | 1777 | 57.1 | 2246 | 0.79 (0.75-0.83) | 64 (50-73) | 51.0% | 39.1% | 95.6% | 13.8% | 13.1% | 14.1% |
| 93C | NHS North Central London CCG | 2911 | 3274 | 57.2 | 3887 | 0.84 (0.81-0.87) | 50 (39-63) | 58.6% | 71.6% | 92.0% | 7.8% | 23.9% | 4.6% |
| 06K | NHS East and North Hertfordshire CCG | 1124 | 1258 | 57.3 | 1498 | 0.84 (0.79-0.89) | 51 (40.2-64) | 59.0% | 26.2% | 86.2% | 7.8% | 13.2% | 3.7% |
| 11X | NHS Somerset CCG | 1206 | 1392 | 63.5 | 1602 | 0.87 (0.82-0.92) | 61 (48-72) | 53.7% | 65.7% | 93.7% | 11.0% | 17.2% | 13.0% |
| 27D | NHS Cheshire CCG | 1555 | 1820 | 62.4 | 2069 | 0.88 (0.84-0.92) | 54 (42-67) | 57.8% | 38.6% | 92.7% | 11.9% | 8.0% | 4.4% |
| M2L0M | NHS Shropshire, Telford and Wrekin CCG | 1104 | 1296 | 65.7 | 1424 | 0.91 (0.86-0.96) | 53 (43-65) | 57.5% | 52.9% | 96.5% | 11.7% | 7.1% | 4.3% |
| 36L | NHS South West London CCG | 3061 | 3606 | 62.0 | 3884 | 0.93 (0.90-0.96) | 50 (39-64) | 61.1% | 68.3% | 93.7% | 11.1% | 9.7% | 6.1% |
| 00V | NHS Bury CCG | 410 | 512 | 67.4 | 546 | 0.94 (0.86-1.02) | 56.5 (44-70) | 50.0% | 61.3% | 89.8% | 15.8% | 10.5% | 8.0% |
| A3A8R | NHS North East London CCG | 3923 | 4629 | 65.4 | 4821 | 0.96 (0.93-0.99) | 49 (38-60) | 58.6% | 64.1% | 94.9% | 12.0% | 13.4% | 4.4% |
| 01Y | NHS Tameside and Glossop CCG | 617 | 767 | 73.7 | 757 | 1.01 (0.94-1.09) | 50 (41-61) | 63.9% | 9.9% | 94.9% | 16.3% | 5.9% | 1.0% |
| 16C | NHS Tees Valley CCG | 1332 | 1804 | 72.7 | 1781 | 1.01 (0.97-1.06) | 55 (43-68) | 56.8% | 41.3% | 94.1% | 22.2% | 7.0% | 2.9% |
| 02P | NHS Barnsley CCG | 615 | 714 | 75.0 | 705 | 1.01 (0.94-1.09) | 48 (37-57.8) | 62.6% | 27.9% | 96.9% | 10.9% | 8.1% | 0.6% |
| 02A | NHS Trafford CCG | 572 | 666 | 71.0 | 657 | 1.01 (0.94-1.09) | 51 (41-64) | 61.6% | 60.5% | 92.0% | 10.7% | 10.2% | 1.7% |
| 00L | NHS Northumberland CCG | 803 | 982 | 75.8 | 967 | 1.02 (0.95-1.08) | 53 (42-65) | 63.3% | 56.8% | 94.3% | 16.2% | 9.5% | 4.2% |
| 05D | NHS East Staffordshire CCG | 323 | 367 | 73.5 | 352 | 1.04 (0.94-1.15) | 56 (44.5-66) | 54.5% | 65.7% | 82.6% | 8.2% | 25.1% | 4.4% |
| 09D | NHS Brighton and Hove CCG | 662 | 801 | 67.1 | 763 | 1.05 (0.98-1.13) | 49 (40-61) | 56.1% | 55.3% | 98.9% | 13.2% | 14.6% | 2.1% |
| 01J | NHS Knowsley CCG | 368 | 465 | 78.4 | 440 | 1.06 (0.96-1.16) | 56 (47-67) | 53.8% | 64.1% | 97.8% | 16.3% | 4.3% | 4.7% |
| 05G | NHS North Staffordshire CCG | 589 | 676 | 74.6 | 638 | 1.06 (0.98-1.14) | 54 (44-67) | 55.5% | 52.4% | 89.5% | 11.7% | 15.1% | 7.5% |
| 06N | NHS Herts Valleys CCG | 1606 | 1737 | 74.7 | 1586 | 1.10 (1.04-1.15) | 52 (41-66) | 60.2% | 65.4% | 89.6% | 5.4% | 25.6% | 3.3% |
| 72Q | NHS South East London CCG | 4130 | 5093 | 75.0 | 4618 | 1.10 (1.07-1.13) | 51 (40-65) | 59.7% | 68.6% | 96.4% | 14.4% | 6.6% | 4.9% |
| 26A | NHS Norfolk and Waveney CCG | 2896 | 3293 | 80.8 | 2999 | 1.10 (1.06-1.14) | 54 (43-67) | 59.2% | 58.5% | 93.2% | 9.4% | 11.8% | 3.8% |
| 02T | NHS Calderdale CCG | 562 | 671 | 79.8 | 607 | 1.10 (1.02-1.19) | 50 (39-63) | 57.5% | 53.4% | 93.1% | 12.8% | 12.5% | 3.6% |
| 92A | NHS Surrey Heartlands CCG | 2710 | 3108 | 75.0 | 2800 | 1.11 (1.07-1.15) | 59 (46-71) | 57.4% | 82.5% | 94.8% | 10.6% | 18.5% | 7.9% |
| 06T | NHS North East Essex CCG | 801 | 1067 | 82.2 | 942 | 1.13 (1.07-1.20) | 60 (46-71) | 54.8% | 59.9% | 96.3% | 23.1% | 8.6% | 3.5% |
| 04Y | NHS Cannock Chase CCG | 379 | 458 | 81.7 | 405 | 1.13 (1.03-1.24) | 53 (42.2-68.8) | 59.6% | 55.2% | 96.1% | 14.8% | 15.3% | 9.0% |
| 00Y | NHS Oldham CCG | 544 | 674 | 79.3 | 599 | 1.13 (1.04-1.21) | 52 (42-65) | 57.3% | 51.8% | 89.3% | 16.0% | 9.2% | 2.4% |
| 15N | NHS Devon CCG | 2996 | 4047 | 83.5 | 3522 | 1.15 (1.11-1.19) | 62 (49-72) | 51.8% | 25.6% | 96.4% | 23.5% | 7.7% | 14.1% |
| D2P2L | NHS Black Country and West Birmingham CCG | 4009 | 4480 | 86.6 | 3713 | 1.21 (1.17-1.24) | 52 (42-65) | 56.2% | 62.5% | 95.4% | 7.5% | 16.8% | 7.3% |
| 99C | NHS North Tyneside CCG | 643 | 754 | 91.8 | 608 | 1.24 (1.15-1.33) | 52 (41-64.8) | 64.6% | 49.7% | 94.8% | 12.1% | 10.1% | 7.8% |
| 42D | NHS North Yorkshire CCG | 1284 | 1560 | 90.2 | 1252 | 1.25 (1.18-1.31) | 57 (45-68) | 57.2% | 34.5% | 90.8% | 15.1% | 26.1% | 4.9% |
| 05V | NHS Stafford and Surrounds CCG | 438 | 566 | 87.6 | 450 | 1.26 (1.16-1.36) | 59 (44-70) | 57.4% | 60.8% | 95.6% | 21.0% | 7.2% | 8.5% |
| 01X | NHS St Helens CCG | 489 | 671 | 92.9 | 534 | 1.26 (1.16-1.36) | 56 (44-66) | 59.5% | 56.9% | 98.1% | 25.0% | 6.6% | 4.6% |
| 02G | NHS West Lancashire CCG | 367 | 439 | 93.1 | 336 | 1.30 (1.19-1.43) | 61 (48.5-71) | 58.3% | 79.3% | 95.2% | 14.1% | 9.6% | 4.6% |
| 99G | NHS Southend CCG | 429 | 699 | 95.6 | 533 | 1.31 (1.22-1.41) | 56 (45-69) | 60.8% | 47.9% | 98.4% | 36.5% | 8.7% | 5.9% |
| 01D | NHS Heywood, Middleton and Rochdale CCG | 666 | 806 | 95.1 | 608 | 1.32 (1.23-1.42) | 55 (41-67) | 54.1% | 62.4% | 88.0% | 13.8% | 11.7% | 4.3% |
| 01V | NHS Southport and Formby CCG | 388 | 482 | 101.0 | 358 | 1.35 (1.23-1.47) | 62 (51-72.8) | 58.5% | 82.6% | 96.3% | 16.0% | 11.2% | 5.2% |
| 99F | NHS Castle Point and Rochford CCG | 415 | 724 | 98.6 | 532 | 1.36 (1.26-1.46) | 55 (45-68) | 60.6% | 46.3% | 98.6% | 39.8% | 7.7% | 7.6% |
| 18C | NHS Herefordshire and Worcestershire CCG | 2679 | 3102 | 98.6 | 2265 | 1.37 (1.32-1.42) | 57 (45-68) | 57.4% | 71.7% | 97.9% | 10.3% | 20.4% | 4.2% |
| X2C4Y | NHS Kirklees CCG | 1493 | 1692 | 97.1 | 1217 | 1.39 (1.32-1.46) | 50 (41-62) | 55.9% | 60.0% | 94.5% | 8.5% | 18.0% | 2.6% |
| 02H | NHS Wigan Borough CCG | 1049 | 1341 | 101.1 | 957 | 1.40 (1.33-1.48) | 55 (43-67) | 59.1% | 62.6% | 97.2% | 19.8% | 5.0% | 3.4% |
| 06L | NHS Ipswich and East Suffolk CCG | 1262 | 1532 | 101.8 | 1088 | 1.41 (1.34-1.48) | 57 (44-68) | 59.3% | 39.1% | 89.0% | 14.8% | 9.4% | 2.9% |
| 05Q | NHS South East Staffordshire and Seisdon Peninsula CCG | 801 | 927 | 101.3 | 655 | 1.42 (1.33-1.51) | 55 (44-67) | 57.4% | 41.2% | 96.1% | 11.4% | 12.3% | 4.6% |
| 03Q | NHS Vale of York CCG | 1219 | 1401 | 95.3 | 973 | 1.44 (1.37-1.52) | 55 (43-68) | 57.5% | 42.8% | 91.1% | 9.4% | 29.1% | 6.0% |
| 12F | NHS Wirral CCG | 1169 | 1455 | 110.7 | 978 | 1.49 (1.41-1.57) | 52 (41-64) | 62.3% | 13.5% | 95.9% | 15.9% | 4.2% | 4.2% |
| 11N | NHS Kernow CCG | 1878 | 2624 | 114.7 | 1739 | 1.51 (1.45-1.57) | 60 (47-69) | 54.8% | 56.3% | 97.9% | 23.8% | 12.8% | 12.6% |
| 02Y | NHS East Riding of Yorkshire CCG | 1333 | 1484 | 112.7 | 975 | 1.52 (1.45-1.60) | 55 (44-67) | 62.2% | 54.0% | 92.4% | 7.7% | 18.1% | 3.2% |
| 13T | NHS Newcastle Gateshead CCG | 1646 | 2023 | 102.6 | 1327 | 1.52 (1.46-1.59) | 51 (40.5-62) | 61.9% | 46.0% | 94.1% | 15.7% | 8.2% | 4.7% |
| 15F | NHS Leeds CCG | 2673 | 2902 | 102.3 | 1878 | 1.54 (1.49-1.60) | 48 (38-60) | 62.2% | 59.8% | 96.7% | 5.5% | 20.5% | 2.0% |
| 02E | NHS Warrington CCG | 659 | 891 | 108.0 | 576 | 1.55 (1.45-1.65) | 56 (43-67.5) | 55.8% | 62.4% | 93.0% | 22.4% | 3.1% | 3.8% |
| 01H | NHS North Cumbria CCG | 1338 | 1517 | 113.2 | 976 | 1.55 (1.48-1.63) | 53 (43-65) | 59.0% | 67.4% | 94.5% | 8.7% | 16.0% | 3.9% |
| 01K | NHS Morecambe Bay CCG | 1273 | 1497 | 110.2 | 959 | 1.56 (1.48-1.64) | 52 (42-64) | 63.1% | 58.4% | 97.0% | 11.8% | 16.5% | 2.0% |
| 36J | NHS Bradford District and Craven CCG | 2175 | 2419 | 109.3 | 1549 | 1.56 (1.50-1.62) | 50 (40-63) | 60.2% | 67.5% | 94.0% | 5.4% | 25.4% | 2.4% |
| 84H | NHS County Durham CCG | 1695 | 2431 | 116.9 | 1543 | 1.58 (1.51-1.64) | 52 (42-62) | 58.8% | 61.5% | 94.2% | 25.8% | 6.1% | 2.6% |
| 03F | NHS Hull CCG | 999 | 1098 | 109.6 | 690 | 1.59 (1.50-1.69) | 48 (36-59) | 60.9% | 55.6% | 91.6% | 7.1% | 16.0% | 2.5% |
| W2U3Z | NHS North West London CCG | 6824 | 8759 | 109.1 | 5473 | 1.60 (1.57-1.63) | 54 (41-66) | 57.5% | 62.7% | 95.9% | 17.6% | 9.5% | 5.8% |
| 01F | NHS Halton CCG | 486 | 630 | 122.5 | 373 | 1.69 (1.56-1.82) | 54 (42-64) | 63.3% | 62.7% | 95.2% | 19.2% | 2.4% | 3.2% |
| 03R | NHS Wakefield CCG | 1581 | 1744 | 126.7 | 997 | 1.75 (1.67-1.83) | 49 (39.8-61) | 63.4% | 67.0% | 99.5% | 6.2% | 15.0% | 2.6% |
| 00Q | NHS Blackburn with Darwen CCG | 613 | 699 | 124.7 | 395 | 1.77 (1.64-1.90) | 49 (38-62) | 60.7% | 62.1% | 94.7% | 7.9% | 27.9% | 1.3% |
| 07K | NHS West Suffolk CCG | 762 | 1038 | 126.8 | 583 | 1.78 (1.67-1.89) | 60 (47-71) | 57.0% | 66.1% | 98.9% | 20.5% | 2.6% | 6.3% |
| 00X | NHS Chorley and South Ribble CCG | 777 | 935 | 134.2 | 492 | 1.90 (1.78-2.03) | 53 (43-66) | 61.2% | 62.0% | 95.1% | 13.2% | 2.9% | 1.5% |
| 02M | NHS Fylde and Wyre CCG | 1006 | 1146 | 142.8 | 597 | 1.92 (1.81-2.03) | 57 (45-68) | 63.6% | 68.8% | 96.6% | 9.0% | 17.4% | 1.2% |
| 15E | NHS Birmingham and Solihull CCG | 4647 | 5946 | 139.9 | 2942 | 2.02 (1.97-2.07) | 54 (43-67) | 56.9% | 36.7% | 98.2% | 18.0% | 5.5% | 6.2% |
| 00R | NHS Blackpool CCG | 720 | 802 | 138.1 | 381 | 2.11 (1.96-2.26) | 52 (40-64) | 59.9% | 70.3% | 95.8% | 6.2% | 19.5% | 3.5% |
| 01A | NHS East Lancashire CCG | 2000 | 2389 | 159.3 | 1085 | 2.20 (2.11-2.29) | 51 (41-64) | 63.5% | 57.8% | 96.9% | 11.8% | 31.9% | 1.0% |
| 01E | NHS Greater Preston CCG | 974 | 1209 | 149.4 | 539 | 2.24 (2.12-2.37) | 54 (43-66) | 64.9% | 54.0% | 95.8% | 16.7% | 3.1% | 1.5% |
| 00N | NHS South Tyneside CCG | 883 | 1381 | 225.3 | 460 | 3.00 (2.85-3.17) | 52 (42-65) | 64.9% | 65.0% | 98.6% | 26.1% | 1.7% | 2.8% |
| 00P | NHS Sunderland CCG | 1851 | 2989 | 265.7 | 822 | 3.64 (3.51-3.77) | 52 (43-64) | 63.6% | 58.4% | 98.3% | 28.8% | 1.4% | 2.3% |
